# Impact of N Incorporation on VLS Growth of GaP(N) Nanowires Utilizing UDMH

**DOI:** 10.1186/s11671-018-2833-6

**Published:** 2018-12-29

**Authors:** Matthias Steidl, Mingjian Wu, Katharina Peh, Peter Kleinschmidt, Erdmann Spiecker, Thomas Hannappel

**Affiliations:** 1 Department of Photovoltaics, Institute of Physics and Institute of Micro- and Nanotechnologies, Technische Universität Ilmenau|, 98693 Ilmenau, Germany; 2 Institute of Micro- and Nanostructure Research & Center for Nanoanalysis and Electron Microscopy (CENEM), Department of Materials Science, Friedrich-Alexander-Universität Erlangen-Nürnberg, 91058 Erlangen, Germany

**Keywords:** III–V nanowires, Dilute nitride, Vapor-liquid-solid growth, Mixed dislocations, Stacking faults

## Abstract

III–V nanowires (NWs) possess great potential for use in future semiconductor technology. Alloying with dilute amounts of nitrogen provides further flexibility in tuning their material properties. In this study, we report on successful in situ nitrogen incorporation into GaP(N) NWs during growth via the Au-catalyzed vapor-liquid-solid (VLS) mechanism. The impact of the nitrogen precursur unsymmetrical dimethyl hydrazine (UDMH) on morphology was found to be overall beneficial as it strongly reduces tapering. Analysis of the crystal structure of NWs with and without N reveals zinc blende structure with an intermediate amount of stacking faults (SF). Interestingly, N incorporation leads to segments completely free of SFs, which are related to dislocations transverse to the growth direction.

## Introduction

III–V nanowires (NWs) have attracted considerable interest as building blocks in almost all fields of semiconductor technology [1–4]. In particular, their small footprint allows for efficient elastic strain relaxation [5] and hence for high crystallinity during heteroepitaxy even if the lattice mismatch is tremendous [6]. This opens a very wide field of material combinations, which are hard to realize with high crystallinity in planar heteroepitaxy. Accordingly, restrictions ruled by the requirement of lattice-matching are reduced, and emphasis can be focused on the engineering of optoelectronical, chemical, and structural properties of the NWs.

Alloying conventional III–V materials with nitrogen constitutes so-called dilute nitride compounds and has been proven to be a strong method to further tailor the material properties [7, 8]. For instance, it leads to a strong reduction of the band gap and a transformation of the indirect band gap of GaP to a quasi-direct one when incorporating more than ca. 0.5% of N [9, 10]. Moreover, dilute amounts of N in GaAs, GaP, and InGaP are reported to significantly improve the chemical stability in aqueous solutions [11, 12], which is of great interest for solar water splitting, where photocorrosion is a serious issue.

N-containing GaP NWs were prepared in the past by sublimation and recondensation of ball-milled GaP powder utilizing NH_3_ as N source [13]. More recently, molecular beam epitaxy (MBE) growth of various different N-containing III–V-core-shell structures has been demonstrated [14–19]. In these studies, commonly a N-free NW core was grown via the vapor-liquid-solid (VLS) growth mode with a Ga droplet as catalyst (known as self-catalyzed growth mode), and subsequently, a dilute nitride shell was grown by conventional layer epitaxy (vapor-solid mechanism). These studies revealed the great potential of dilute nitride NWs and discovered beneficial properties related to their architecture, such as decreased surface recombination [20], increased light harvesting via energy up-conversion [21], and emission of linearly polarized light [22, 23].

Nevertheless, dilute nitride materials continuously suffer from strong non-radiative recombination, an issue which is known to be closely related with the formation of defects, such as interstitials, antisites, vacancies, and impurity atoms [24–27]. Their formation in turn strongly depends on conditions and parameters applied during growth. For example, hydrogen appears to promote the formation of point defects [28], and the choice of precursors and epitaxy method has a significant impact on defect formation [26, 29]. Since the VLS-growth of NW (cores) significantly differs from vapor solid growth of layers (or shells), the density of detrimental point defects might be reduced applying the VLS growth mechanism. So far, VLS growth of dilute nitrides was only achieved by self-catalyzed growth [18, 19], which is however restricted by small growth windows. Therefore, parameters have to be carefully tuned and well-defined doping is very challenging [30, 31]. Moreover, this growth mode frequently struggles with parasitic island growth and inhomogeneous NW dimensions [18, 19]. In contrast, Au-catalyzed VLS NW growth is very versatile and rather easy to control and allows for precisely tunable and high doping levels [1, 31–33]. First attempts reported in the literature to prepare dilute nitride NWs via Au-catalyzed VLS growth have, however, not been successful as the N-precursor suppressed one-dimensional growth [34].

In this study, we demonstrate successful dilute nitrogen incorporation via the Au-catalyzed VLS-growth mechanism. We find incorporation of N on group V sites and an overall advantageous impact on morphology and crystal structure by the utilization of the nitrogen precursor unsymmetrical dimethyl hydrazine (UDMH).

## Methods

GaP(N) NWs were grown by the Au-catalyzed vapor-liquid-solid (VLS) growth mode on GaP(111)B substrates via metalorganic vapor phase epitaxy (MOVPE, Aixtron AIX 200). Only liquid precursors were used with trimethylgallium (TMGa), tertiarybutylphosphine (TBP), and unsymmetrical dimethylhydrazine (UDMH) as precursors for Ga, P, and N, respectively. Prior to NW growth, the substrates were cleaned in acetone and isopropyl alcohol and deposited with monodisperse Au particles from colloidal solution. Annealing at 550 °C under overpressure of TBP was carried out for 15 min, in order to desorb the surface oxide and form liquid Au-Ga droplets. Subsequently, NWs were grown with a TMGa molar fraction of χ_TMGa_ = 6.16 × 10^−5^ and a TBP/TMGa ratio of 10. The applied growth temperatures range from 500 to 550 °C, and UDMH:TBP ratios between 0:1 (i.e., pure GaP) and 9:1 were investigated. If not explicitly stated otherwise, the duration of growth was 16 min and the Au-particle size 50 nm. During the whole process, the reactor pressure was 50 mbar with a total gas flow of 3.4 l/min, which was provided by H_2_ as carrier gas. All specified temperatures were measured by a thermocouple within the graphite suszeptor.

The samples were characterized by means of high-resolution scanning electron microscopy (SEM, Hitachi S 4800-II). Two of the samples were selected for microscopic and spectroscopic investigation with transmission electron microscopy (TEM). The TEM samples were mechanically dry-transferred on lacey carbon grids. TEM studies were performed on a ThermoScientific Titan^3^ Themis operating at 200 kV. The microscope is equipped with ultra-bright X-FEG electron source and spherical aberration correctors in both illumination and imaging sides. Electron energy loss spectra were recorded using the attached GIF Quantum ERS in diffraction mode with a collection angle of ~ 3 mrad, which is optimized for detection of the N-K edge at 403 eV. For Raman spectroscopy, the NWs were transferred by the same means on Si substrates. A green 532-nm laser with 400 μW was used as the excitation source and focused with a × 50 objective. The signal was analyzed with a cooled Si charge-coupled device (CCD) detector.

## Results and Discussion

### Morphology

**Fig. 1:**
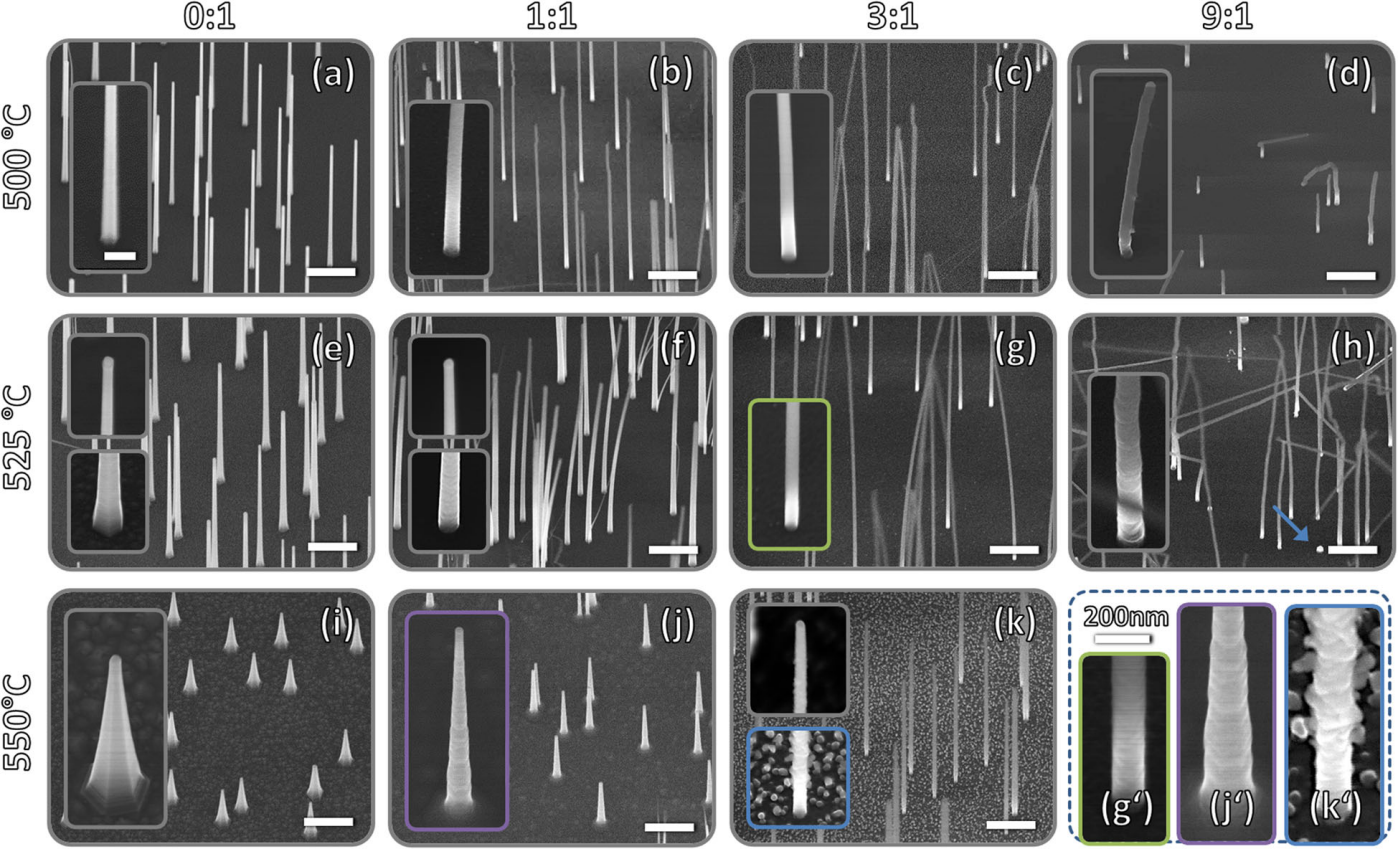
VLS-grown GaP(N) nanowires on GaP(111)B. UDMH:TBP ratio and temperature were varied from 0 to 9 and 500 to 550 °C, respectively. The growth time was always 16 min. All overview and close-up scans were taken at 30° tilt and have the same scale, respectively, with the measuring bars being 2 μm or 200 nm. In (g′), (f’), and (k’) enlarged close-ups are shown for a clear visibility of the surface

In Fig. 1, the morphologies of differently prepared GaP(N) NWs are shown. Note that the bending and touching of NWs with very high aspect ratio was not present right after growth, but is due to electrostatic attraction during SEM investigation [35]. The same effect additionally leads to a distortion at the top of some NWs (cf Fig. 1b, c).

The microscopic images reveal that for all parameters investigated in this study, growth of freestanding NWs has been achieved. Moreover, in most cases, all NWs are straight and vertical to the substrate as well as homogenous in length. In contrast to self-catalyzed dilute nitride NWs [14, 18, 19], no parasitical island growth was observed. These NW properties are considered essential to match the common demands for their use in applications. Besides, it can be seen that both the temperature and the UDMH concentration (expressed as UDMH:TBP ratio) have a tremendous impact on the NW morphology: increasing the temperature leads to a length reduction and enhances parasitic vapor-solid (VS) growth on the NW side facets. Both effects intensify NW tapering. Tapering is generally undesired, as the parasitic shell can degrade the functionality of devices for reasons of geometry, different compositions [36], and/or doping levels or even doping directions [37]. From the equal growth duration of all samples follows that, the axial growth rate (GR) decreases with temperature, while the coaxial GR increases. The impact of increasing UDMH concentrations, in contrast, is generally beneficial: with increasing UMDH ratio the axial GR rises, while the radial GR declines. Hence, tapering is drastically reduced—particularly for higher temperatures. Apart from that, very high UDMH ratios of 9:1 lead to unstable growth conditions. This instability is reflected in a frequent change of the growth direction and a wide length dispersion—partially, NW growth is even completely suppressed (see arrow in Fig. 1h). Another feature of NW growth with high UDMH ratios is surface roughening, which is exacerbated both by higher temperature and higher UDMH supply (compare images g′, j′ ,and k′). At 550 °C and a concentration of 3:1 (k and k′), where the surface is the roughest, it becomes evident that the roughening decreases from bottom to top and does not occur immediately below the Au particle. This proves that this effect is not related to the VLS growth but to the parasitic shell growth instead. The reason for this roughening might be strain due to a strong and possibly inhomogeneous incorporation of nitrogen [38] into the shell.

**Fig. 2:**
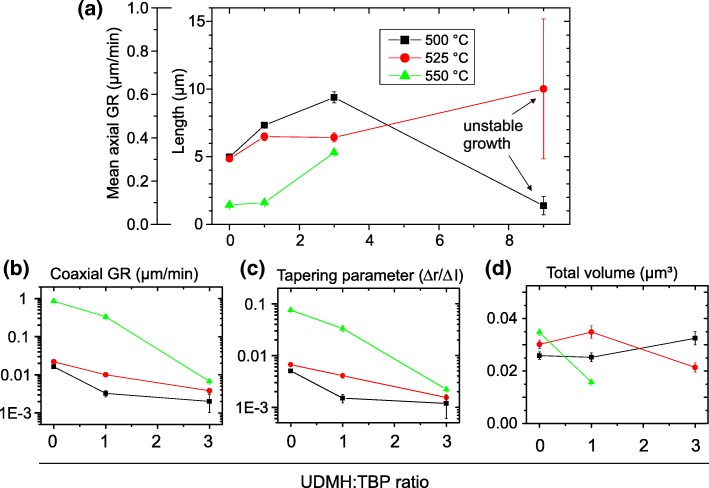
Geometric characteristics of the NWs from Fig. 1 as a function of growth temperature and UMDH:TBP ratio: (**a**) length and mean axial growth rate, (**b**) coaxial growth rate, (**c**) tapering parameter, (**d**) total volume. Each measurement point represents an averaging of 10 to 20 NWs with the error bar representing the standard deviation or error propagation. The mean total volume of a NW in (**d**) was estimated assuming a truncated cone with circular cross-section

An evaluation of the geometric characteristics of the NWs, which is presented in Fig. 2, illustrates the tendencies described above. While the axial GR (a) increases with supply of UDMH and decreases with temperature, it is the direct opposite for the coaxial GR (b). Accordingly, the tapering parameter (c), which is defined as the difference of the radius at the top and the bottom divided by the NW length, is low for high UDMH ratios and low temperatures. Note that this definition of the tapering parameter is equal to the ratio between coaxial and axial GR.

This increase of tapering with temperature is a prevalent phenomenon in NW growth and can be explained as follows [39]: At low temperatures (≤ 500 °C), VS growth is kinetically limited, while VLS growth is only limited by the mass transport of the growth species. As the temperature increases, the kinetic barrier of VS growth is increasingly traversed, so that the coaxial GR rises. Since the VS and VLS growth compete for material, the temperature increase causes a simultaneous decrease in the axial GR. This effect can be further enhanced by an increased desorption rate and the concomitant reduction of the diffusion length. As TMGa is already fully pyrolyzed at 450 °C [40] and it is TMGa which limits the GR at V/III = 10, decomposition kinetics should play a minor role. It should be noted though that generally, besides temperature, the III–V ratio and absolute precursor flow have a tremendous impact on growth kinetics, so that tapering free NWs can be achieved also at high temperature (see, e.g., [41] for WZ-GaAs NWs and [42] for InP-NWs).

In the following, the decrease in tapering caused by the addition of UDMH is discussed. From Fig. 2a and b, it is evident that it is both due to accelerated axial VLS growth and decelerated coaxial VS growth. A similar impact on the GR is observed for the addition of HCl [33, 43] or tert-butyl-chloride (TBCl) [44] during NW growth. In both cases, the VS growth on the side facets is reduced or completely suppressed by a corrosive effect of the chlorine species [45–47]. At the same time, the axial GR increases (at least for low HCl or TBCl concentrations). It is argued that the portion of the group III species, which would contribute to VS growth in the absence of the Cl species, contributes to VLS growth instead, probably in the form of InCl for InP NWs [33]. While in these studies, the increase of Cl species always involves a decrease in the NW volume [33, 44], the volume of the GaP(N) NWs investigated here depends comparatively little on the concentration of UDMH and in some cases even increases with the UDMH concentration (Fig. 2d). For this reason, an etching effect of UDMH is very unlikely. Instead, UDMH and its fragments could sterically hinder VS growth on the side facets. There is strong evidence that a large amount of UDMH and its fragments are present as adsorbates on the NW side facets between 500 °C and 550 °C. This evidence includes the following points: first, the incomplete decomposition of UDMH, which should be proceeded by only about 5% to 30% between 500 and 550 °C [48–51]; secondly, the high concentration of UDMH in the gas phase, which equals 10 to 90 times the amount of TMGa; thirdly, experiments with in situ spectroscopy on GaPN layers, which indicate that UDMH and its fragments attach to the surface after growth and cooldown (below 650 °C), whereas this is not the case for TBP and its fragments [52]. These adsorbates prevent the Ga species from reaching the NW facets and contributing there to VS growth. Instead, they diffuse to the Au particle where they promote VLS growth. VLS growth will be significantly less affected by steric hindrance, since the surface of the Au particle acts as collector and measures about 3 times the growth front (interface between Au particle and NW). In addition, a catalytic effect of Au [53, 54] may favor the pyrolysis of UDMH and thereby promote the removal of the more volatile fragments.

### Raman Spectroscopy

In order to investigate nitrogen incorporation and structural properties, Raman spectroscopy was carried out on individual NWs in the back scattering geometry. The NWs analyzed with Raman spectroscopy differ from the NWs shown in Fig. 1, in that they have a larger diameter (100 nm) and were grown only for 8 min. This ensures that the influence of parasitical VS overgrowth is negligibly small. For example, for a UDMH ratio of 3:1, the shell’s percentage of the cross-sectional area in the middle of the wire (where measurements were carried out) is less than 3%. As a reference, a lattice-matched GaP_1 − *x*_N_*x*_ layer on Si (100) with *x* = 2.1% was measured, too. All spectra are normalized with respect to the longitudinal optical (LO) mode of GaP.

All spectra exhibit GaP-like transversal optical phonon modes (TO_Γ_) at 365 cm^−1^ and longitudinal optical phonon modes (LO_Γ_) at 399–403 cm^−1^, which are based on Raman scattering at phonons in the center of the Brillouin zone (Γ point). In addition, spectral components near 387 cm^−1^ (X), at 397 cm^−1^ (SO), around 500 cm^−1^ (NLVM), and the LO mode of the Si substrate (LO^Si^) at 522 cm^−1^ were observed. The 750–820 cm^− 1^ range contains modes from second-order Raman scattering (SORS).

At low UDMH:TBP ratios (0.1 and 0.3), surface optical (SO) phonons at 397 cm^−1^ are observable [55–57]. This surface activated phonon mode can arise from diameter modulation [55], rough surfaces [56], and/or structural defects [57]. With increasing UDMH ratios, the SO mode either vanishes or gets superimposed by a mode referred to as X (sometimes denoted as LO_X_). Its occurrence is commonly explained by a break of the translational symmetry [58–60], which in our case will be caused by insertion of N into the GaP matrix. This causes relaxation of the momentum conservation rules and thereby allows zone-boundary longitudinal optical phonon scattering due to phonons at or near the X point [59, 61]. As the X mode steadily increases with the UDMH ratio, it can be concluded that the incorporation rises, too [61–63]. Unfortunately, the intensity of the X mode does not allow for a quantification of the N content, since its exact relation with the N content is unknown and strongly depends on measurement conditions. In contrast, the intensity of the N-related local vibrational mode (NLVM) at ~ 500 cm^− 1^ scales almost linearly with the concentration of (substitutional) nitrogen *x*, if *x* ≤ 2.1% and spectra are normalized to the LO mode [58]. Since the NLVM is caused by vibrations of Ga–N bonds, it only reflects substitutional nitrogen [62, 64, 65]. Note that NLVM sometimes is denoted as LO_2_. With the planar GaPN_0.021_ reference being measured under the same conditions, the substitutional N concentration of the GaP(N) NWs can be determined from the NLVM/LO_Γ_ area ratio. Due to the overlapping LO mode of Si, peak deconvolution has to be applied. It yields NLVM/LO_Γ_(GaPN) = 0.44 ± 0.03 and NLVM/LO_Γ_(NW,3:1) = 0.145 ± 0.028. Accordingly, a substitutional N-concentration of x_3:1_ = (0.7 ± 0.2)% for an UDMH:TBP ratio of 3:1 is determined. For lower UDMH ratios, however, the intensity of the NLVM is too low for quantification.

As mentioned in the introduction, previous attempts by Suzuki et al. to incorporate N during Au-catalyzed VLS growth (of GaAs(N) NWs) failed [34]. Even if the reasons can be manifold, we consider the growth sequence to be the greatest difference (with respect to our study) and, thus, the most likely source for the failure. Suzuki et al. applied pulsed-jet epitaxy, where each of the precursors is offered separately for several seconds (referred to as pulse). Since for VLS growth the species have to travel a longer distance compared to layer growth and incorporation into the crystal is delayed through the liquid seed particle, mass transport and desorption will play a critical role. In this context, also the type of precursor and its decomposition kinetics will be crucial—as we observe in our study (cf. Figure 2).

Furthermore, increasing UDMH ratios lead to an enhancement of second-order Raman processes (SORS). This is remarkable because for planar GaPN the opposite is the case. There, N incorporation causes a strong quenching and broadening of SORS peaks [58]. This is a consequence of the high sensitivity of second-order scattering processes to lattice distortion on the scale of few lattice constants (compared to first order scattering) [58, 66]. Probable sources of such lattice distortions are N clusters and local distortion due to the short and stiff Ga–N bond [58]. Conversely, this indicates that the lattice distortion in NWs decreases with increasing UDMH concentration despite enhanced nitrogen incorporation. This could be related to a reduction of stacking faults upon UDMH supply, as both the interplanar spacing increases with hexagonality (i.e., SF density) [67] and periodicity is disturbed by each SF. Note that the normalization of the spectra on the LO_Γ_ is excluded as potential origin, since prior normalization the intensity of LO_Γ_ was about two to three times larger for N-containing structures.

### TEM and EELS

In order to verify this conclusion, transmission electron microscopy (TEM) was conducted on NWs grown with and without supply of UMDH. Moreover, EELS was applied as a complementary method to prove N incorporation.

**Fig. 3:**
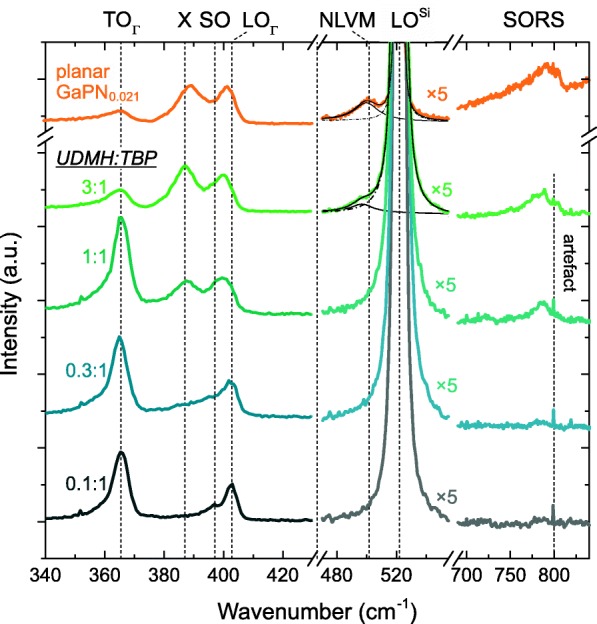
μ-Raman spectra of GaP(N) NWs grown with UDMH:TBP ratios ranging from 0.1 to 3. A lattice-matched GaPN layer on Si acts as reference (orange). For the deconvolution of the NLVM component Pseudo Voigt functions (of same shape) were used. The sharp line at 800 cm^−1^ is a measurement artefact

**Fig. 4:**
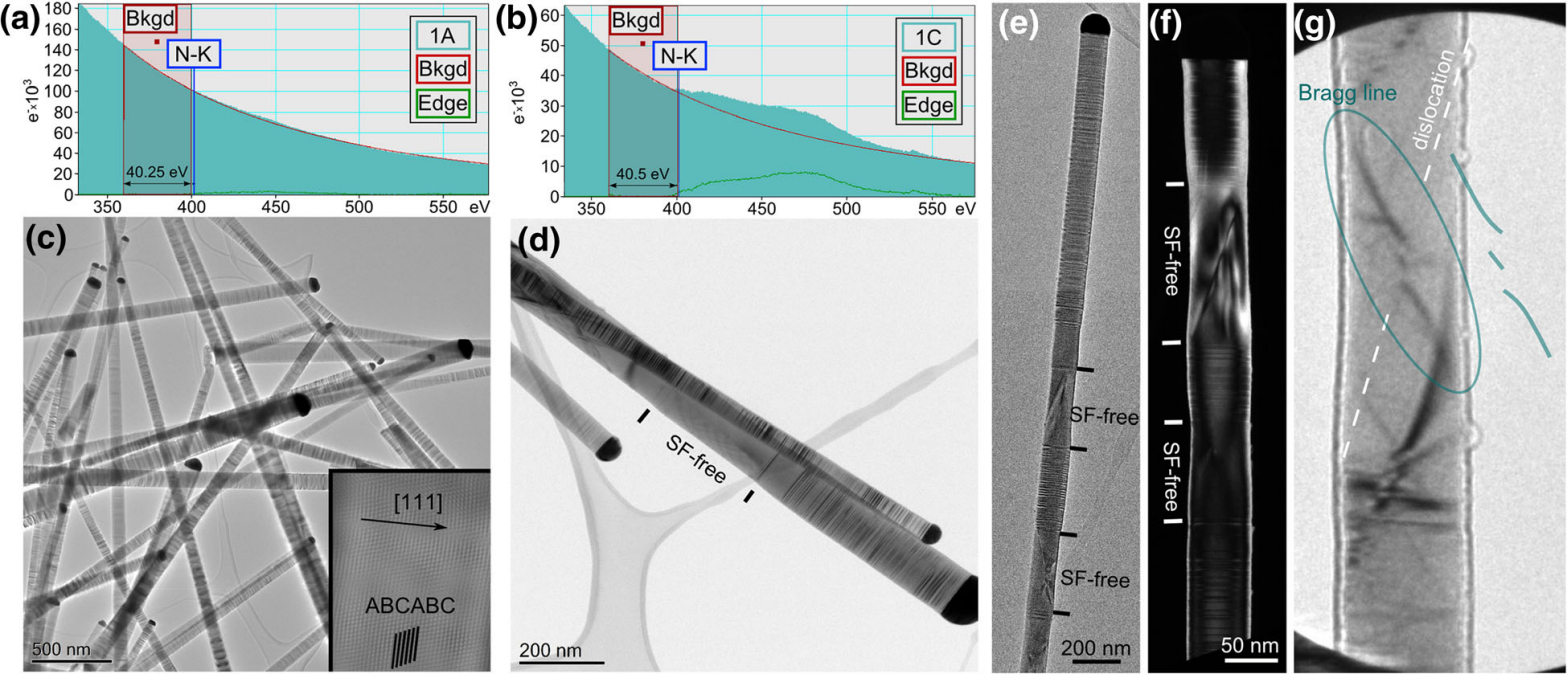
TEM results of sample 1A and 1C, grown without and with supply of UDMH, respectively. The designation follows the panel names in Fig. 1. Electron energy loss (EEL) spectrum of sample 1A (**a**) and 1C (**b**), the incorporation of N in sample 1C is clearly revealed. TEM micrographs of sample 1A (**c**) and 1C (**d**). The inset in (**c**) is a Fourier filtered HRTEM image of a small SF-free region in sample 1A. Despite that the sample is a couple of degrees off the ⟨110⟩ zone axis, the ABCABC stacking of GaP is still visible, confirming the zincblende structure. SF-free sections in sample 1C are highlighted. Bright-field (**e**) and dark-field (**f**) TEM images show strong strain contrast along the line of the diagonal of SF-free region. Typical twist and splitting of Bragg line [63] in the large-angle convergent beam electron diffraction (LACBED) pattern in (**g**) confirms the presence of dislocation in the SF-free region (highlighted in dark cyan)

Figure 4 summarizes the TEM studies on the samples: sample 1A was grown without UDMH supply and sample 1C was grown with a UDMH:TBP ratio of 3—both samples were prepared at 500 °C. The designation follows the panel names in Fig. 1. In the EEL spectrum, the N-K edge at 400 eV is clearly seen in sample 1C, while hardly detectable in sample 1A (cf. Fig. 3a b). Both samples exhibit predominant zinc blende (ZB) structure, as can be seen from the ABCABC stacking in Fourier filtered HRTEM images of a NW luckily oriented close to the ⟨110⟩ zone axis (but still a couple of degrees off, cf. inset in Fig. 4c for sample 1A). Rather high densities of SFs between 150 and 200 μm^−1^ can be seen in both samples. Strikingly, in sample 1C, SF-free sections of typically 150–300 nm in length can frequently be observed. Considering the similar SF densities for N-free and the N-containing NWs, it appears that it is the SF-free segments which give rise to the enhancement of the SORS processes with increasing UDMH concentrations (cf. Fig. 3).

Bright-field (BF) and dark-field (DF) imaging with various g-vectors of such SF-free segments reveal a strong strain field of single dislocations running diagonally from one end of the SF-rich region to the other (cf. Fig. 4e, f). In large angle convergent beam electron diffraction (LACBED, cf. Fig. 4g), a typical twist and splitting of the Bragg line is observed when encountering the defect line. This proves that it is indeed a dislocation, i.e., a line defect, and not a planar defect, e.g., an inclined plane boundary, because such a planar boundary would result in a shift of the Bragg line in LACBED and not in the observed twist and splitting [68]. From the inclination angle of the dislocation line, and BF-TEM images with g.b visibility criteria, the dislocations are mixed-type comprising screw and edge character. Considering that SFs are grown-in defects and that the dislocation is pinned between SFs, it is likely that the dislocation has also formed during growth and not afterwards caused by mechanical stress. This conclusion is additionally strengthened by the slightly reduced diameter within the SF-free regions. Most probably, the dislocation formation is caused by high local strain due to N incorporation and the very different bond lengths of Ga–N and Ga–P.

A likely explanation for the absence of stacking faults by the presence of a dislocation is given in the following. It is known that VLS growth normally proceeds via layer-by-layer growth, where 2D nuclei determine whether the next layer will follow the stacking sequence (ZB nucleus) or forms SF (WZ nucleus). For Au-catalyzed growth, under most conditions, nuclei form at the triple phase boundary [39]. In this case, nucleation barriers of ZB and WZ are very close, leading to frequent SF formation, as also observed in the investigated NWs.

With the presence of a dislocation, the growth mechanism is significantly altered, leading to preferred incorporation of material in the vicinity of the dislocation core. Here, the two characters of a dislocation have to be considered. The screw character gives rise to protruding atoms (out of the flat {111} surface) acting as preferred incorporation sites for atoms transforming from liquid to solid. The spiral arrangement of atoms along the dislocation line (cf. Refs. [69, 70]) dictates the stacking sequence, which is defined when the dislocation nucleates. Only when the dislocations runs out, stacking disorder is again possible. The reason why the dislocation exists still inside the nanowire is likely the anisotropic stress field in the radial direction of its edge component, which is compressive at one side and tensile at the other one. This gives rise to a net force dragging the dislocation during growth towards the center, and finally to the other edge of the NW in a diagonal manner. The straight course of it is a result of the dislocation line tension.

## Conclusion

We have shown how to incorporate dilute amounts of nitrogen into GaP NWs during Au-catalyzed VLS growth and have demonstrated impacts on the crystalline structure of GaP(N) NWs. Raman spectroscopy proves increasing amounts of N with rising supply of the nitrogen precursor UDMH and verifies incorporation at group V sites. Studying a wide range of UDMH concentrations and temperatures, we found an overall advantageous impact of UDMH on the morphology. This is reflected in reduced NW tapering, which we attribute to steric hindrance of incompletely pyrolized UDMH molecules. TEM analysis reveals zinc blende structure in both N-free and N-containing NWs with a rather high stacking fault (SF) density. Strikingly, N-containing NWs exhibit 150–300 nm long regions without any SFs, which are interspersed with individual dislocations. It seems that these dislocations are formed during NW growth and suppress SF nucleation. This study demonstrates the suitability of the common N-precursor UDMH for N incorporation in VLS-grown NWs and will enable further tailoring of the NW material properties.

## Abbreviations

BF: Bright-field
DF: Dark-field
EELS: Electron energy loss spectroscopy
GR: Growth rate
LACBED: Large angle convergent beam electron diffraction
MBE: Molecular beam epitaxy
MOVPE: Metalorganic vapor phase epitaxy
NLVM: Nitrogen-related local vibrational mode
NW: Nanowire
SEM: Scanning electron microscopy
SF: Stacking fault
SORS: Second-order Raman scattering
TBP: Tertiarybutylphosphine
TEM: Transmission electron microscopy
TMGa: Trimethylgallium
UDMH: Unsymmetrical dimethyl hydrazine
VLS: Vapor-liquid-solid
VS: Vapor-solid
WZ: Wurtzite
ZB: Zinc blende
